# Prevalence and Associated Risk Factors of Human Intestinal Protozoan Parasitic Infections in Ethiopia: A Systematic Review and Meta-Analysis

**DOI:** 10.1155/2020/8884064

**Published:** 2020-10-05

**Authors:** Dires Tegen, Destaw Damtie, Tamirat Hailegebriel

**Affiliations:** ^1^Dera Woreda Education Office, South Gondar Zone, Ethiopia; ^2^Department of Biology, College of Sciences, Bahir Dar University, Bahir Dar, Ethiopia

## Abstract

**Background:**

Human intestinal protozoan parasitic infections (HIPPIs) are a series of public health problems in developing countries like Ethiopia. The overall prevalence of HIPPIs in Ethiopia is not known. Therefore, this systematic review and meta-analysis study is aimed at determining the overall prevalence of HIPPIs in Ethiopia.

**Methods:**

Articles written in English were searched from online public databases. Searching terms used were “prevalence,” “intestinal protozoan parasite,” “associated factors,” and “Ethiopia.” We used Stata version 14 for meta-analysis and Cochran's *Q* test statistics and the *I*^2^ test for heterogeneity.

**Result:**

A total of 286 articles were reviewed, but only 45 of them fulfilled the inclusion criteria. The pooled prevalence of HIPPIs in Ethiopia was 25.01% (95% CI: 20.08%-29.95%) where *Entamoeba histolytica*/*dispar* is the most prevalent (14.09%, 95% CI: 11.03%-17.14%) followed by *Giardia lamblia* (10.03%, 95% CI: 7.69%-12.38%) and *Cryptosporidium* spp. (5.93%, 95% CI: 2.95%-8.91%). This meta-analysis showed that family size (OR: 3.7, 95% CI: 1.45-5.85), source of drinking water (OR: 3.33, 95% CI: 1.30-5.36), open field defecation (OR: 2.91, 95% CI: 1.60-4.21), handwashing habit (OR: 2.82, 95% CI: 2.01-3.63), playing with soil (OR: 2.15, 95% CI: 1.01-3.29), the habit of eating raw vegetables (OR: 1.77, 95% CI: 1.03-2.51), and fingernail trimming (OR: 1.70, 95% CI: 0.89-2.25) were strongly associated with the HIPPIs in Ethiopia. High heterogeneity on the prevalence of HIPPIs was observed among studies within and among regions (*I*^2^ > 99% and *P* ≤ 0.01).

**Conclusion:**

The prevalence of HIPPIs was significantly high among the Ethiopian population. Family size, source of drinking water, open field defecation, handwashing habit, the habit of eating raw vegetables, and fingernail trimming habits were significantly associated with HIPPIs.

## 1. Background

Parasitic infection is one of the major health problems where more than 3.5 billion people are infected globally. Parasitic infections result in 450 million and 200,000 annual morbidities and mortalities, respectively [1]. Protozoan infections are among such infections. They are highly prevalent in preschool children in developing countries [2, 3]. *E*. *histolytica*/*dispar*, *G*. *lamblia*/*duodenalis*, and *Cryptosporidium* spp. are the major common pathogenic intestinal protozoan species globally reported [4].

*E*. *histolytica* has an annual incidence rate of five million cases, affects approximately 500 million people worldwide, and results in 50 million annual symptomatic diseases and 100,000 deaths [5]. It too results in 2.2 million disability-adjusted life years [6, 7]. *G*. *lamblia*/*duodenalis* infects 280 million people annually. It results in two and half million cases of diarrhea every year in resource-poor countries alone. In these countries, the prevalence of giardia infection is acquired during early infancy and it reaches up to 30% in children younger than 10 years of age [8]. The global prevalence of cryptosporidiosis is 1 to 4.5% in developed countries and 3 to 20% in developing countries. Its infection rate in AIDS patients ranges from 3 to 20% in the United States and 50 to 60% in Africa and Haiti [9]. It results in an estimate of 8.37 million DALYs with cryptosporidiosis [10].

Transmission of *E*. *histolytica*, *G*. *lamblia*/*duodenalis*, and cryptosporidiosis is through the oral-fecal route following direct or indirect contact with the infectious stages, including human-to-human, zoonotic, waterborne, and foodborne transmission [11]. However, *Cryptosporidium* may also be transmitted airborne [12]. Eating unwashed fruits, nail-biting, sucking fingers, and contact with infected family members are also key factors contributing to the increased HIPPIs [13, 14].

Protozoan infections are serious public health concerns and are responsible for iron deficiency anemia, growth retardation, and physical and mental health problems among children. They also lead to nutritional depletion, poor immunity in infants, mucosal loss and lymphatic leakages, and local hemorrhages [2].

Despite remarkable development in medical science in recent years, protozoan parasitic infections remain serious health issues in developing countries like Ethiopia [15]. Low level of environmental sanitation, personal and food hygiene, contamination of water with human excreta, and lack of awareness about simple health promotion practices such as personal hygiene and food hygiene make HIPPIs the most common problems in Ethiopia [16–19]. The prevalence of HIPPIs in Ethiopia is different in different parts of the country. However, there is no summarized pooled overall prevalence of HIPPIs. Therefore, this systematic review and meta-analysis study is aimed at producing the pooled prevalence and factors associated with HIPPIs from available studies in Ethiopia.

## 2. Methods

### 2.1. Study Design and Setting

Ethiopia is located in the horn of Africa. It is bounded by Eritrea to the north, Djibouti and Somalia to the east, Sudan and South Sudan to the west, and Kenya to the south. Currently, the Ethiopian population is estimated to be 113,620,337 with 21.3% (24,463,423) people living in the urban area and a median age of 19.5 years. Ethiopia's population is equivalent to 1.47% of the total world population. The population density in Ethiopia is 115/km^2^ (298 people/mi^2^) [20]. The total land area is 1,104,300 km^2^ [21].

### 2.2. Search Strategies

Articles written in English were searched from online public databases, namely, PubMed/MEDLINE, ScienceDirect, Web of Science, Google Scholar, Hinari, WorldCat, and Cochrane Library [22], using core search terms and phrases: “prevalence,” “intestinal protozoan parasite,” “associated factors,” and “Ethiopia.” The search terms were used separately and in combination using Boolean operators like “OR” or “AND.” Besides, Gray literature was searched through the review of available references. Searching of pieces of literature included in this meta-analysis was conducted from December 2019 to January 2020.

### 2.3. Inclusion and Exclusion Criteria

Studies that were written in the English language, reporting about the prevalence of HIPPIs and their associated risk factors in Ethiopia, published from 2008 to January 2020, with sample sizes above 100 [23] were included in the study. Studies conducted on HIV/AIDS patients, meta-analysis, and review articles were excluded from this systematic review and meta-analysis study.

### 2.4. Data Extraction

The data extraction protocol was prepared and evaluated by all authors. The data extraction protocol consists of the name of the author and year of publication, region, nature of study subjects (school-age children, food handlers, preschool-age children, patients, rural dwellers, and street dwellers), total sample size, number of positive cases, estimated prevalence, species of intestinal parasites, and potential risk factors associated with individual species of HIPPIs.

### 2.5. Quality Assessment of Individual Studies Included in the Meta-Analysis

The Grading of Recommendations Assessment, Development and Evaluation (GRADE) approach was used to assess the overall quality of evidence [24]. The quality of each study was declared using the three major assessment tools (methodological quality, comparability, and outcome and statistical analysis of the study). Two points were given to each criterion. Publications with a total score of 5–6 points were considered to be high, 4 points to be moderate, and 0–3 points to be low-quality publications [24]. The association between HIPPIs and associated risk factors was calculated in the form of the log odds ratio. The odds ratio was calculated for the common associated risk factors of the reported studies.

### 2.6. Risk of Publication Bias across Studies Included in This Meta-Analysis

The risks of publication bias across studies were assessed using funnel plot symmetry and Egger's test. Egger's test (*P* value < 0.05) was used to determine the presence of publication bias across studies.

### 2.7. Outcomes of the Study

Intestinal protozoan parasitic infection and associated risk factors of HIPPIs were the two major outcome variables.

### 2.8. Data Analysis

The pooled prevalence of HIPPIs was calculated by dividing the total positive cases to the total study subjects included in this meta-analysis. Cochran's *Q* test and *I*^2^ statistics were used to assess heterogeneity among the studies [25]. There was a clear heterogeneity on the prevalence of HIPPIs across studies included in this meta-analysis; we used a random-effects model to estimate the pooled effect size. To sort out the causes of heterogeneity, we conduct a subgroup analysis based on the region of the study, the nature of study participants, study year, and sample size included in individual studies. The presence of publication bias was assessed using Egger's test and symmetry of the funnel plot. The cause of publication bias was assessed using a sensitivity test and regression test. Forest plot format was used to present the pooled point prevalence with 95% CI. A log odds ratio was used to decide the association between associated risk factors and HIPPIs among respondents included in the studies. The meta-analysis was conducted using Stata software (version 14, StataCorp, College Station, TX), where *P* < 0.05 was considered statistically significant.

## 3. Results

**Figure 1:**
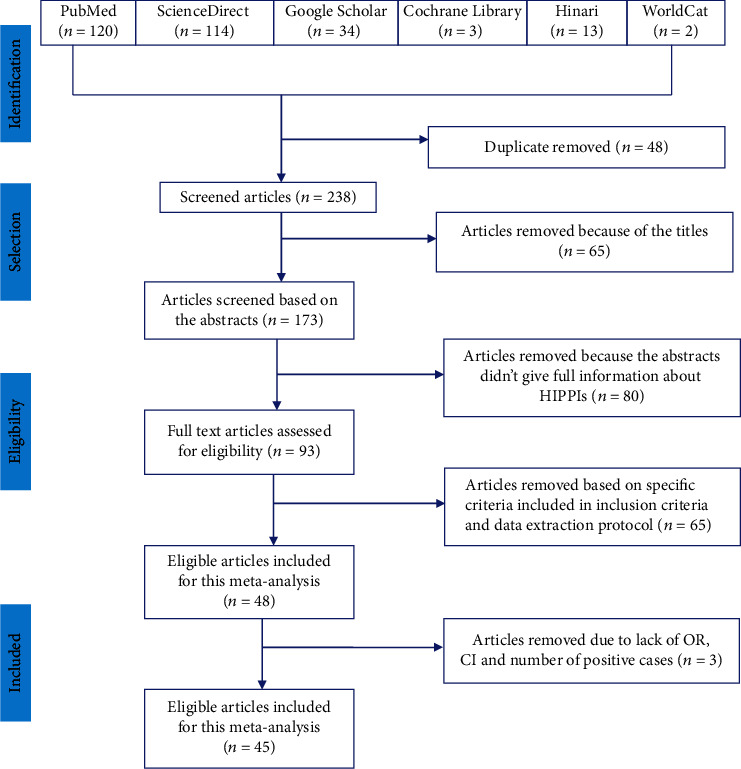
PRISMA flow diagram of articles considered for the review on HIPPIs among the Ethiopian population.

A total of 286 articles on the prevalence and associated risk factors of intestinal parasitic infections in Ethiopia were retrieved. Forty-eight of these articles were excluded due to duplicates. From the remaining 238 articles, 65 were excluded after review of their titles (titles were not related to HIPPIs) and 80 were excluded after review of abstracts (lack of full information about protozoan parasites). The remaining 93 full-text articles were accessed and assessed for eligibility based on the inclusion criteria and information indicated in the data extraction protocol. As a result, 48 articles were further excluded in the data extraction process primarily due to the outcome of interest, and they did not have OR, 95% CI, and the number of positive cases (meaning the report was only based on estimated prevalence percent). Thus, only 45 (15.7%) of the studies met the eligibility criteria and were included in the final systematic review and meta-analysis study (Figure 1).

### 3.1. Characteristics of Original Studies Included in the Meta-Analysis

Out of the 45 screened studies, 44 (97.5%) of them were cross-sectional and one (2.5%) was a case-control study. A total of 131,916 study participants were involved, and the sample size of the studies ranged from 127 to 89,423. Twenty (44.44%) of the studies were from Amhara, 8 (17.77%) from SNNPR, 6 (13.33%) from Oromia, 2 (4.44%) from Addis Ababa, 1 (2.22%) from Dire Dawa, 2 (4.44%) from Benishangul-Gumuz, and 6 (13.33%) from Tigray regions (Table 1). Unfortunately, there were no studies reported from Afar, Harari, Gambela, and Somali regional states of Ethiopia.

### 3.2. Quality of Studies Included in the Meta-Analysis

The quality score of each original study ranged between three and the highest six (Table 1). The overall quality of the articles included in this meta-analysis is very good.

### 3.3. Prevalence of Intestinal Protozoan Parasitic Infections in Ethiopia

**Figure 2:**
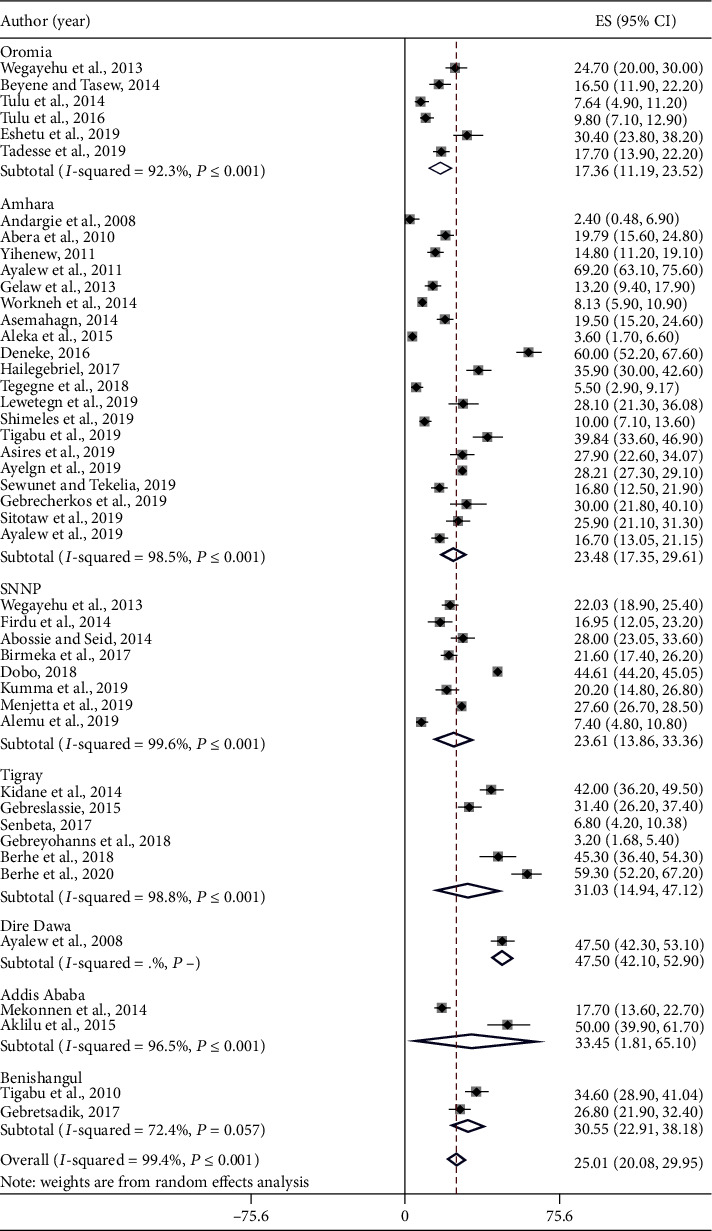
Pooled prevalence of HIPPIs in Ethiopia including the regional level. Each square represents the effect size (ES) of individual studies, and the horizontal line represents the 95% CI. The diamond indicates the pooled effect, and the vertical dash lines indicate the overall estimate.

The lowest prevalence of IPPIs was reported from Gondar (2.4%) [26] while the highest prevalence (69.2%) was reported from Delgi primary school, Amhara region [27]. The overall pooled prevalence of HIPPIs was 25.01% (95% CI: 20.08%-29.95%) (Figure 2). High heterogeneity (*I*^2^ = 99.4%, *P* ≤ 0.001) was observed across studies included in the studies.

### 3.4. Subgroup Analysis

High pooled prevalence of HIPPIs was reported from Dire Dawa (47.5%, 95% CI: 42.10-52.9), followed by Addis Ababa (33.45%, 95% CI: 1.81-65.10), Tigray (31.03%, 95% CI: 14.94-47.12), and Benishangul-Gumuz (30.55%, 95% CI: 22.91-38.18), whereas the low prevalence of HIPPIs was observed in Oromia (17.35%, 95% CI: 11.17-23.53) followed by Amhara (23.48%, 95% CI: 17.35-29.61) and SNNPR (23.61%, 95% CI: 13.86-33.36). The pooled prevalence of HIPPIs of studies with sample sizes > 200 (24.89%, 95% CI: 19.86-29.93) was lower than that of studies having sample sizes ≤ 200 (27.12%, 95% CI:-2.2-56.45) (Table 2).

The highest pooled prevalence of HIPPIs was observed among patients (32.65%, 95% CI: 25.64-39.67) followed by school children (24.21%, 95% CI: 17.89-30.52), food handlers (22.24%, 95% CI: 12.94-31.54), urban dwellers (17.7%, 95% CI: 13.15-22.25), preschool children (15.81%, 95% CI: 2.23-29.40), and rural dwellers (13.29%, 95% CI: 0.85-25.72) (Table 2).

The pooled prevalence of HIPPIs was 31.28% (95% CI: 12.78-49.77), 25.30% (95% CI: 18.33-32.27), and 22.58% (95% CI: 17.52-27.64) observed among studies conducted from 2008 to 2012, 2018 to 2020, and 2013 to 2017, respectively (Table 2).

### 3.5. Common HIPPIs in Ethiopia

**Figure 3:**
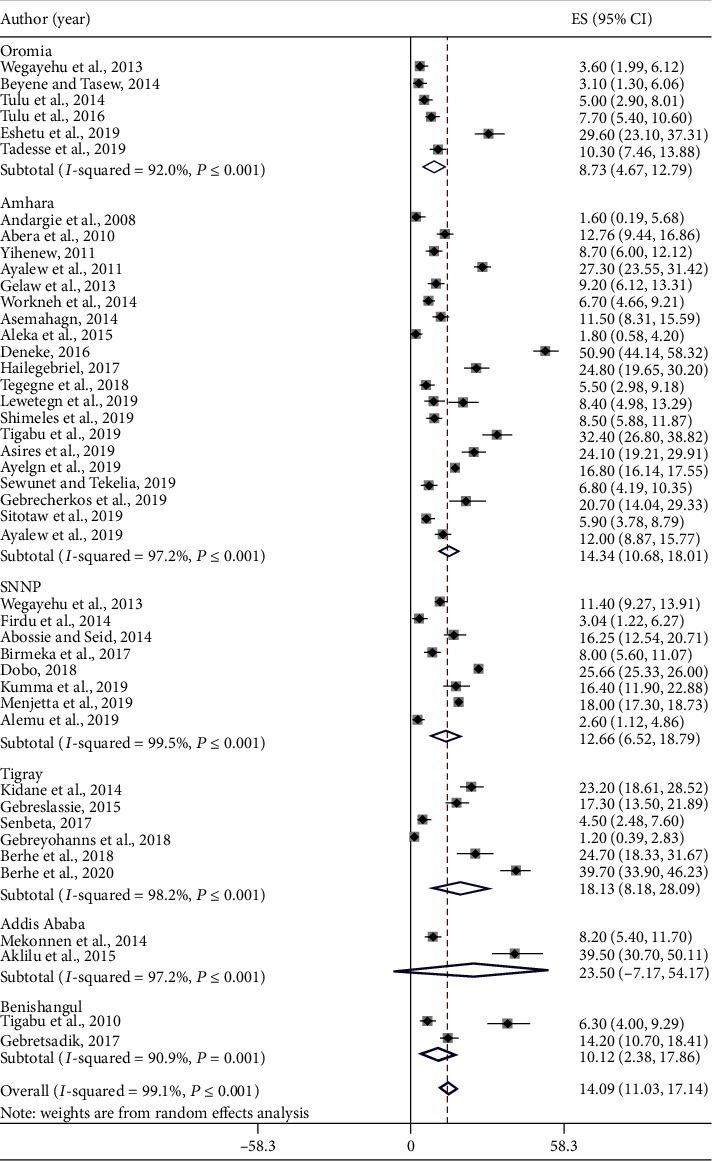
The pooled prevalence of *E*. *histolytica*/*dispar* among study participants in Ethiopia.

**Figure 4:**
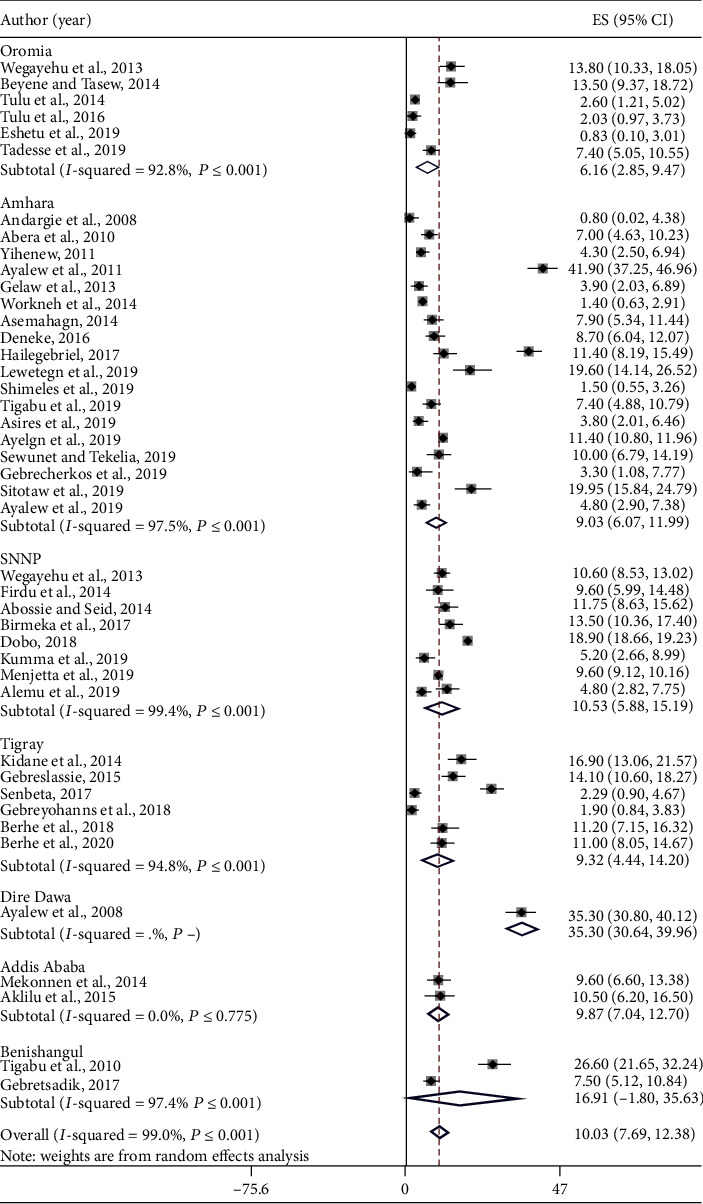
The pooled prevalence of *G*. *lamblia* among study participants in Ethiopia.

**Figure 5:**
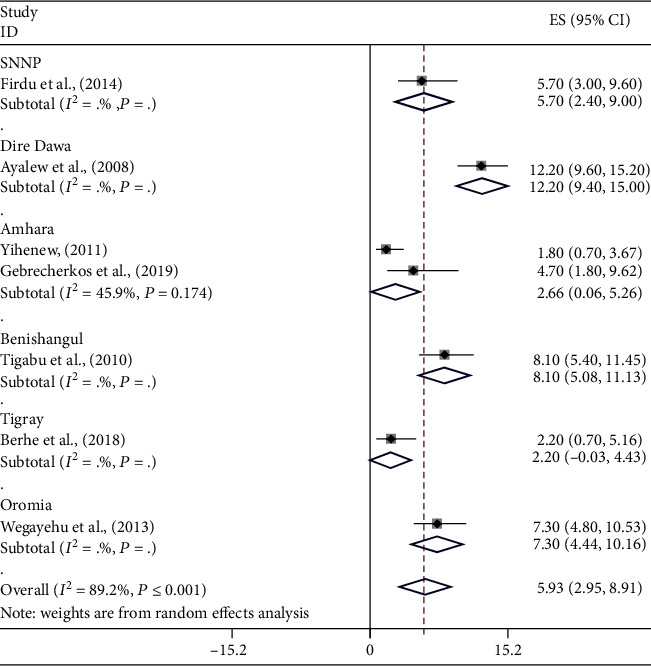
The pooled prevalence of *Cryptosporidium* spp. among study subjects in Ethiopia.

The pooled prevalence of *E*. *histolytica*/*dispar* was 14.09% (95% CI: 11.03-17.14) (Figure 3) followed by *G*. *lamblia* 10.03% (95% CI: 7.69-12.38) (Figure 4) and *Cryptosporidium* spp. 5.93% (95% CI: 2.95-8.91) (Figure 5) among study subjects in Ethiopia. There is no significant difference in the pooled prevalence of *E*. *histolytica*/*dispar*, *G*. *lamblia*, and *Cryptosporidium* spp. among regions included in this meta-analysis (*P* = 0.322, *P* = 0.168, and *P* = 0.088, respectively).

We perform a metaregression analysis to identify the sources of heterogeneity across studies. The analysis showed that the region of study (regression coefficient: 0.104, 95% CI: -0.032-0.239, and *P* = 0.131), nature of study subjects (regression coefficient: -0.158, 95% CI: -0.322-0.006, and *P* = 0.058), year of publication (regression coefficient: -0.029, 95% CI: -0.365-0.306, and *P* = 0.861), and sample size (regression coefficient: 0.041, 95% CI: -1.024-0.941, and *P* = 0.933) did not contribute for the heterogeneity.

### 3.6. Risk of Publication Bias across Studies Included in the Meta-Analysis

**Figure 6:**
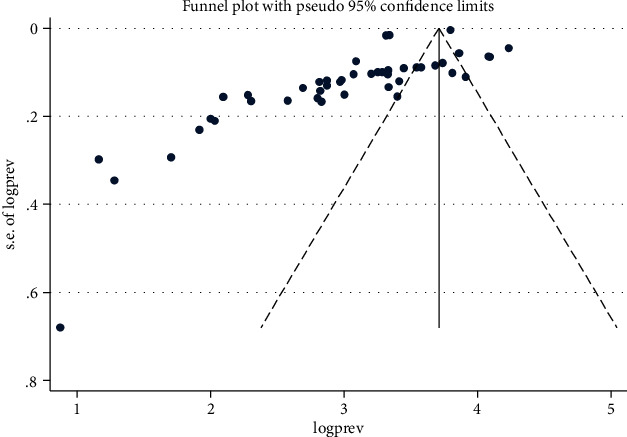
Funnel plot indicates publication bias among studies.

The funnel plot symmetry demonstrates the presence of publication bias among studies included in this meta-analysis (Figure 6). Similarly, Egger's test results (*P* ≤ 0.001) indicate publication bias among studies. Sensitivity analysis was performed by recalculating the pooled prevalence of HIPPIs by sequentially removing one by one to identify the cause of publication bias. The pooled prevalence remained stable, and the result was not driven by individual studies included in the meta-analysis.

### 3.7. Factors Associated with HIPPIs in Ethiopia

In this meta-analysis, we have reviewed several potential risk factors associated with HIPPIs in Ethiopia. Family size, source of drinking water, open field defecation, handwashing habit, the habit of eating raw vegetables, and fingernail trimming and cleanness habits were associated with HIPPIs.

The association between fingernail trimming and cleanness habits and intestinal protozoan parasitic infection in people of Ethiopia was computed from 12 studies [18, 28–38]. The pooled results showed that individuals with poor fingernail trimming and cleanness habits were 1.7 times more likely to be infected with HIPPIs than their counterparts (OR: 1.7, 95% CI: 0.89-2.25, and *P* ≤ 0.001) (Supplementary 1 = S1).

The pooled results of 20 studies [17, 18, 27, 28, 31–34, 36–47] showed that handwashing habits were strongly associated with infection with intestinal protozoan parasitic infections in Ethiopia.

The odds of having an intestinal parasitic infection was 2.82 times higher among people who did not wash their hands after defecation than people who wash their hands (OR: 2.82, 95% CI: 2.01-3.63) (S2).

The association between age and intestinal protozoan parasitic infections was analyzed from nine studies [18, 29, 31, 34, 41, 42, 47–49]. Age (children up to 14 years) was significantly associated with the prevalence of intestinal protozoan parasitic infections. The odds of having HIPPIs in children (up to 14 years) were 1.54 times higher than those in adults (OR: 1.54, 95% CI: 0.91-2.18) (S3).

The association between open field defecation and intestinal protozoan parasitic infection among people in Ethiopia was computed from six studies [3, 34, 35, 38, 42, 50]. People who practiced open field defecation were 2.91 times more likely to have intestinal protozoan parasitic infections than those who did not practice open field defecation (OR: 2.91, 95% CI: 1.60-4.21) (S4).

The association between the habits of eating raw, unwashed, contaminated, leftover fruits and vegetables with intestinal protozoan parasitic infections was evaluated from nine studies [18, 27–29, 34, 35, 38, 44, 45]. The odds of having intestinal protozoan parasitic infections was 1.77 times higher among people who had habits of eating raw, unwashed, contaminated, leftover fruits and vegetables as compared with the counterparts (OR: 1.77, 95%; CI: 1.03-2.51) (S5).

The association between family education and intestinal protozoan parasitic infection among people in Ethiopia was computed from ten studies [3, 17, 27–29, 31, 42, 46, 48, 50]. The pooled results showed that uneducated mothers, fathers, and children were 1.69 times more likely to have intestinal protozoan parasitic infections than those who were educated (OR: 1.69, 95% CI: 0.84-2.54) (S6).

Results from six studies revealed that the level of household income was strongly associated with HIPPIs [31, 33, 41, 42, 44]. People who had low household income were 1.64 times more likely to have intestinal protozoan parasitic infections than those who had higher family income (OR: 1.64, 95% CI: 0.96-2.32) (S7).

The pooled results of eleven studies [17, 27, 28, 30, 31, 38, 42, 46, 47, 50, 51] revealed that people who drink unprotected water were 3.33 times more likely to have intestinal protozoan parasitic infections than those who drink protected water (OR: 3.33, 95% CI: 1.30-5.36) (S8).

The pooled analysis of two studies conducted in Ethiopia [3, 35] showed that people who had the habit of playing with soil were 2.15 times more likely to have intestinal protozoan parasitic infections than those who did not (OR: 2.15, 95% CI: 1.01-3.29) (S9).

The pooled results of five studies [31, 45–47, 50] showed that family sizes were strongly associated with intestinal protozoan parasitic infection among people in Ethiopia. The likelihood of intestinal parasitic infection was 3.7 times higher among people with a family size of above 5 that those with low family sizes (OR: 3.7, 95% CI: 1.45-5.85) (S10).

## 4. Discussion

Human intestinal protozoan infections are the major IPIs and are the common causes of morbidity and mortality in Ethiopia [23]. Knowing the exact national pooled prevalence of HIPPIs is useful for policymakers. The pooled prevalence of HIPPIs in this systematic review and meta-analysis study was 25.01% (95% CI: 20.08-29.95). It was higher than studies conducted in Côte d'Ivoire (18.7%) [52], Tanzania (17.4%) [53], Saudi Arabia (18.7%) [54], and Qatar (5.93%) [55]. However, it was almost similar to the studies from Bulgaria (25.53%) [56], Spain (28%) [57], the Democratic Republic of São Tomé and Príncipe (28.6%) [58], and Iran (21%) [59].

The prevalence of HIPPIs in the present study was lower than that of Libya (85%) [14]; Shahura Health Center, Amhara region, Ethiopia (39.84%) [49]; Cambodia (53.9%) [58]; Senegal (32.6%) [60]; Thailand (37.8%) [61]; Palestine (39.21%) [62]; Ghana (42.9%) [63]; Sudan (54.2%) [64]; Tripoli, Kenya (56%) [65]; Kut city, Iraq (57.5%) [66]; Iraq (98.8%) [67]; Cameroon (74.3%) [68]; Malaysia (72.3%) [69]; Mexico (65%) [70]; Mexico (60%) [71]; and Burkina Faso (84.7%) [72]. The differences may be attributed to methodological, social, economic, demographic, hygienic, weather and climatic, environmental, and political factors.

The highest HIPPI prevalence was observed among patients (32.65%, 95% CI: 25.64-39.67) and schoolchildren (24.21%, 95% CI: 17.89-30.52) while the lowest prevalence was observed among rural dwellers (13.29%, 95% CI: 0.85-25.72) and preschool children (15.81%, 95% CI: 2.23-29.40). Parental care among preschool children may be the reason for their lower prevalence. But school-age children have higher prevalence due to their habit of playing with soil [3, 35]. The high prevalence of HIPPIs among patients might be due to their high susceptibility to protozoan parasites that might be associated with low immunity. Intestinal parasitic infections were more prevalent among the poor divisions of the population with poor handling of personal and environmental sanitation [46].

The trend of HIPPIs in Ethiopia from 2008 to 2012, 2013 to 2017, and 2018 to 2020 were 31.28% (95% CI: 12.78-49.77), 22.58% (95% CI: 17.52-27.64), and 25.30% (95% CI: 18.33-32.27), respectively. Accordingly, the prevalence of HIPPIs in the first ten years was reduced from 31.28% to 22.58%. However, it rose from 2018 to 2020 by close to 2.72%. This finding disagreed with the study done in Qatar in which HIPPI prevalence reduced from 2005 to 2008 (7.98%, 95% CI: 7.429-8.536), 2009 to 2011 (5.13%, 95% CI: 4.673-5.593), and 2012 to 2014 (4.89%, 95% CI: 4.488-5.286) [55]. The rise of HIPPIs from 2018 to 2020 in this study may be due to a lack of mass treatment, especially school deworming [73].

The pooled prevalence of *G*. *lamblia* was 10.03% in this meta-analysis, which is in line with the studies conducted in Côte d'Ivoire (13.1%) [52], Tanzania (10.6%) [53], Ghana (12.2%) [63], Iraq (10.8%) [67], and Nepal (12.5%) [74]. However, it was lower than studies in Tripoli, Libya (28.5%) [14]; Bulgaria (62.05%) [56]; Spain (18%) [57]; Cambodia (31.5%) [58]; Senegal (20.4%) [60]; Sudan (22.9%) [64]; Mexico (24%) [70]; Burkina Faso (28.1%) [72]; Dhaka (17.6%) [75]; the Philippines (19.2%) [76]; and Turkey (47.97%) [77]. But, *G*. *lamblia* prevalence (10.03%) in this study was much higher than that of Saudi Arabia (3%) [54], Thailand (4.2%) [61], Kenya (6.5%) [65], Iraq (4%) [66], Cameroon (3.3%) [68], Iran (1.7%) [59], Libya (4.9%) [78], Turkey (6.1%) [79], and Thailand (0.6%) [80]. The variations might be due to variations in the quality of drinking water sources and environmental conditions [81].

The pooled prevalence of *E*. *histolytica*/*dispar* was 14.09% in the present meta-analysis. It agrees with the reports from Pahang, Malaysia (18.5%) [69]; the Philippines (12.1%) [76]; and Karnataka, India (9%) [82]. However, the result of this study was higher than that of studies in Saudi Arabia (2%) [54], Iran (0.6%) [59], Thailand (0.73%) [61], Ghana (0.21%) [63], Cameroon (7.3%) [68], and Mexico (5%) [70]. In contrast, the results of the present study were lower than those of studies from Côte d'Ivoire (56%) [52]; Tanzania (28.5%) [53]; Sudan (31.2%) [64]; Kenya (23.9%) [65]; Kut city, Iraq (41%) [66]; Iraq (88%) [67]; Burkina Faso (66.5%) [72]; and Libya (21.14%) [78]. The variation might be due to the quality of food and water and the environmental condition of the different study localities. *E*. *histolytica*/*dispar* is an environmental contaminant of drinking water supply and food. It can be transmitted by drinking infected water and by consuming contaminated vegetables and food [81].

The pooled prevalence of *Cryptosporidium* species was 5.93%, which is higher than other studies from Saudi Arabia (3%) [54], Bulgaria (1.69%) [56], Spain (1%) [57], Iran (0.4%) [59], Senegal (0.3%) [60], Dhaka (0.5%) [75], Libya (0.8%) [78], and China (2.4%) [83]. The result of this meta-analysis was lower than that of studies conducted in Iraq (10.4%) [14], Ghana (8.5%) [63], Kenya (13%) [65], Iraq (12.5%) [66], Cameroon (44%) [68], and the Philippines (22%) [76]. The reason for the low prevalence of *Cryptosporidium* infection in Ethiopia might be associated with the types of laboratory diagnostic procedures. About 84% studies included in this meta-analysis did not use appropriate methods for detecting opportunistic parasites such as *Cryptosporidium*.

This meta-analysis and systematic review study showed that there was a clear variation in the prevalence of HIPPIs in Ethiopia. The highest pooled prevalence was observed from Dire Dawa while the lowest prevalence was obtained from the Oromia region. The potential justification for this difference might be due to the peculiarity in sociodemographic, environmental, geographical, and behavioral characteristics. Similar observations were reported from Nepal [84].

The odds of HIPPI occurrence was 1.7 times higher among groups who did not have regular fingernail trimming and cleanness habits compared to their counterparts. This finding is supported by the studies conducted in Sri Lanka [13] and Nepal [74]. It is known that unclean fingernails may contain cysts of protozoan parasites that lead to higher infection.

The habit of handwashing was significantly associated with the prevalence of HIPPIs in Ethiopia. The odds of having HIPPIs among people who did not wash their hands after defecation was about 2.8-fold higher than that among people who used to wash their hands regularly. This finding is opposed to the studies conducted in Sudan [64] and Nepal [74]. This might be due to fecal-oral contamination through unwashed hands.

Age (children up to 14 years) was significantly associated with the prevalence of intestinal protozoan parasitic infections. The result was supported by the studies conducted in Iran [59], Palestine [62], Ghana [63], Sudan [64], Cameroon [68], Libya [78], and Karachi [85]. This might be because children have weak immunity than adults. Their complex nutritional requirements and less developed immune systems make children the principal sufferers of the intestinal parasitic infections [1].

The habit of eating raw, unwashed, contaminated, and leftover fruits and vegetables was significantly associated with intestinal protozoan parasitic infections. This was supported by the studies done in Sri Lanka [13] and Tripoli, Libya [14]. This is because raw, unwashed, contaminated, and leftover fruits and vegetables carry intestinal protozoan parasites [81].

Families with low-level education were 1.3 times more likely to have HIPPIs than educated families. This result was supported by studies done in Iran [59], Cameroon [68], Mexico [70], and Nepal [74]. The reason may be uneducated people lack the necessary knowledge and practices towards the transmission and prevention of HIPPs [70].

In this meta-analysis, drinking of unprotected water was significantly associated with the occurrence of HIPPIs in Ethiopia. The people who drink unprotected water were 3.3 times more likely to have intestinal protozoan parasitic infections than those who use protected water. This finding was in line with the studies done in Ethiopia [46], Sudan [64], Cameroon [68], Mexico [71], Turkey [77], and China [83]. This is because unprotected water would have a pool of intestinal protozoan parasites and can be a source of infection. Water is one of the important vehicles for pathogen dissemination [86]. *E*. *histolytica*/*dispar*, *Cryptosporidium,* and *G*. *lamblia* are environmental contaminants of drinking water supplies [82].

This meta-analysis showed that open field defecation was significantly associated with the presence of human intestinal protozoan parasitic infections. People who practiced open field defecation were 2.74 times more likely to have intestinal protozoan parasitic infections than their counterparts. The result of this study was in accordance with the study done in Ethiopia [46], Mexico [70], and South India [87]. In developing countries, poor hygiene and the use of untreated human feces are important factors that contribute to the contamination of food and water. Due to this, *E*. *histolytica*, *G*. *lamblia*, and *Cryptosporidium* spp. could be transmitted to humans [88]. Intestinal parasitic infections also occur via contaminated material such as earth, water, uncooked, or cross-contaminated food that has been in contact with the feces of an infected individual or animal [89].

People who had poor levels of income were 1.64 times more likely to have intestinal protozoan parasitic infections than their counterparts. This might be due to lack of treatment, medication, and quality food and water and poor living conditions among the people who had poor economic levels. This result is in agreement with the study from Mexico [70]. On the contrary, this finding was opposed to the study done in Gondar, Ethiopia; the level of income is not associated with the prevalence of intestinal parasitic infections [41].

The people who had the habit of playing with soil were about 2-fold more likely to have HIPPIs than their counterparts. The study was in accordance with the study conducted in North Shewa, Ethiopia [3]. This is because the soil contains eggs and cysts of protozoan intestinal parasites which could contaminate food and water [5].

The people who had a large family size (>5) were nearly 4-fold more likely to have intestinal protozoan parasitic infections than those who have a small family size. This might be because a large family size would increase the chance of more contact with each other and could be a source of transmission of protozoan infections [2]. The outcome was in line with studies done in Ethiopia [46] and Turkey [77].

## 5. Limitations of the Study

This meta-analysis and systematic review study produced a lot of valuable data about intestinal parasites in Ethiopia, but it has also several limitations. The articles included in this meta-analysis were not derived from all regions (information about HIPPIs was lacking from Afar, Gambela, Somali, and Harari regions). Besides, the information used in this meta-analysis was not uniformly distributed in the regions included in this meta-analysis. Therefore, the result may not fully represent the national prevalence of intestinal parasitic infection.

## 6. Conclusion

The pooled prevalence of intestinal protozoan parasitic infections according to this review was found to be 25.01%. There was a clear difference in the prevalence of HIPPIs across regions in the country. Fingernail trimming, handwashing habits, age, open field defecation, the habit of eating raw fruits and vegetables, level of family education, levels of income, source of drinking water, playing with soil, and family size were significantly associated with the prevalence of intestinal protozoan parasitic infections. This study highlights the importance of proper health education on personal hygiene, handwashing practice, open field defecation, handling of food, selections of living rooms of animals to prevent animal contact, and food safety. Therefore, all stakeholders should give proper attention to increasing awareness of the community and proper treatments of infected patients.

## Figures and Tables

**Table 1:** Characteristics of the study subjects included in the eligible articles used for this review.

Source	Year	Region	Study area	Sample size	Cases	Prevalence (95% CI)	Quality score
Andargie et al.	2008	Amhara	Gondar food handler	127	3	2.4% (0.48-6.90)	4
Ayalew et al.	2008	Dire Dawa	Lege Dini watershed	655	311	47.50% (42.30-53.10)	3
Abera et al.	2010	Amhara	Bahir Dar food handler	384	76	19.79% (15.60-24.80)	6
Tigabu et al.	2010	Benishangul	Pawi	384	133	34.6% (28.90-41.04)	6
Ayalew et al.	2011	Amhara	Delgi primary school children	704	487	69.20% (63.10-75.60)	6
Yihenew	2011	Amhara	Fogera Awuramba	392	58	14.8% (11.20-19.10)	3
Gelaw et al.	2013	Amhara	Gondar health center	304	40	13.20% (9.40-17.90)	6
Wegayehu et al.	2013	Oromia	North Shewa Zone children	384	95	24.70% (20.00-30.00)	6
Wegayehu et al.	2013	SNNPR	Gamo rural residence	858	189	22.0% (18.90-25.40)	6
Abossie and Seid	2014	SNNPR	Chencha town primary school	400	112	28% (23.05-33.60)	6
Andualem	2014	Amhara	Motta Town primary school	364	70	19.50% (15.20-24.60)	6
Beyene and Tasew	2014	Oromia	Jimma health center	260	43	16.50% (11.90-22.20)	5
Firdu et al.	2014	SNNPR	Yirgalem health center	230	39	16.95 (12.05-23.20)	6
Kidane et al.	2014	Tigray	Wukro Town health center	384	163	42% (36.20-49.50)	6
Mekonnen et al.	2014	Addis Ababa	Addis Ababa	355	63	17.7% (13.60-22.70)	6
Tulu et al.	2014	Oromia	Delo-Mena, Bale Zone	340	26	3.60% (4.90-11.20)	6
Workneh et al.	2014	Amhara	Debre Elias primary school	541	44	8.13% (5.90-10.90)	6
Aklilu et al.	2015	Addis Ababa	Addis Ababa University	172	86	50% (39.90-61.70)	3
Aleka et al.	2015	Amhara	Gondar University Hospital	277	10	3.6% (1.70-6.60)	6
G/Silassie et al.	2015	Tigray	Aksum Town primary school	404	127	31.4% (26.20-37.40)	4
Deneke	2016	Amhara	Ankober	403	240	60.0% (52.20-67.60)	3
Tulu et al.	2016	Oromia	Dolomena (Balie Zone)	492	48	9.8% (7.10-12.90)	6
Birmeka et al.	2017	SNNPR	Gurage zone primary school	450	97	21.60% (17.40-26.20)	6
Gebretsadik	2017	Benishangul	Homsha district	395	106	26.8% (21.90-32.40)	6
Hailegebriel	2017	Amhara	Bahir Dar	359	129	35.9% (30.00-42.60)	6
Senbeta	2017	Tigray	Adigrat primary school	309	21	6.80% (4.20-10.38)	5
Berhe et al.	2018	Tigray	Mekele	226	101	45.3% (36.40-54.30)	5
Dobo	2018	SNNPR	Hawasa	89423	39895	44.6% (44.20-45.05)	3
Gebreyohanns et al.	2018	Tigray	Addiremets town	411	13	3.2% (1.68-5.40)	5
Tegegne et al.	2018	Amhara	Gondar University Hospital	256	14	5.5% (2.90-9.17)	5
Alemu et al.	2019	SNNPR	Gamogofa Zone primary school	351	26	7.4% (4.80-10.80)	6
Asires et al.	2019	Amhara	East and West Gojjam	344	96	27.9% (22.60-34.07)	5
Ayalew et al.	2019	Amhara	Bahir Dar primary school	418	71	16.98% (13.05-21.15)	5
Ayelgn et al.	2019	Amhara	Gondar poly health center	13329	3760	28.2% (27.30-29.10)	5
Eshetu et al.	2019	Oromia	Nekemit	240	73	30.4% (23.80-38.20)	6
Gebrecherkos et al.	2019	Amhara	University of Gondar	150	45	30% (21.80-40.10)	6
Kumma et al.	2019	SNNPR	Wolayta Sodo University	233	47	20.2% (14.80-26.80)	6
Lewetegn et al.	2019	Amhara	Senbete and Bete Towns	214	60	28.1% (21.30-36.08)	6
Menjetta et al.	2019	SNNPR	Hawasa University Clinic	13679	3782	27.6% (26.70-28.50)	4
Sewunet and Tekelia	2019	Amhara	Woreta	310	52	16.8% (12.50-21.90)	5
Shimeles et al.	2019	Amhara	Chagni food handler	400	40	10% (7.10-13.60)	6
Sitotaw et al.	2019	Amhara	Jawi primary school	406	105	25.9% (21.10-31.30)	6
Tadesse et al.	2019	Oromia	Bamo no. 2 primary school	417	74	17.7% (13.90-22.20)	5
Tigabu et al.	2019	Amhara	Shahura health center	364	145	39.84% (33.60-46.90)	6
Berhe et al.	2020	Tigray	Adigrat	418	248	59.30 (52.20-67.20)	6

SNNPR: Southern Nations, Nationalities, and People's Region.

**Table 2:** Prevalence of HIPPIs in Ethiopia by subgroups.

Variables	Characteristics	Number of studies	Sample size	Prevalence (95% CI)	*I* ^2^, *P* value
Sample size	≤200	3	449	27.12% (95% CI: -2.2-56.45)	99.4%, *P* < 0.01
	>200	42	131,467	24.89% (95% CI: 19.86-29.93)	97.8%, *P* < 0.01

Pooled prevalence of HIPPIs by year	2008-2012	6	2646	31.28% (95% CI: 12.78-49.77)	99.0%, *P* < 0.01
	2013-2017	20	7681	22.58% (95% CI: 17.52-27.64)	96.7%, *P* = 0.01
	2018-2020	19	121,589	25.30% (95% CI: 18.33-32.27)	99.6%, *P* < 0.01

Nature of study participants	Food handlers	7	1900	22.24% (95% CI: 12.94-31.54)	96%, *P* < 0.01
	Patients	13	118,738	32.65% (95% CI: 25.64-39.67)	99.%, *P* < 0.01
	Preschool children	3	858	15.81% (95% CI: 2.23-29.40)	96%, *P* < 0.01
	School children	18	8404	24.21% (95% CI: 17.89-30.52)	97.7%, *P* < 0.01
	Urban dwellers	1	355	17.7% (95% CI: 13.15-22.25)	—
	Rural dwellers	3	1661	13.29% (95% CI: 0.85-25.72)	98.1%, *P* < 0.01

Overall		45	131,916	25.01% (95% CI: 20.08-29.95)	99.4%, *P* < 0.01

## Data Availability

All related data has been presented within the manuscript and on supplementary data. The dataset supporting the conclusions of this article is available from the authors on request.

## Abbreviations

AOR: Adjusted odds ratio
CDC: Center for Disease Controlling program
CI: Confidence interval
DALYs: Disability-adjusted life years
GRADE: Grading of Recommendations Assessment, Development and Evaluation
HIPPIs: Human intestinal protozoan parasitic infections
HIPPs: Human intestinal protozoan parasites
IPIs: Intestinal parasitic infections
OR: Odds ratio
PRISMA: Preferred Reporting Items for Systematic Reviews and Meta-Analyses
spp.: Species
WHO: World Health Organization.

## Supplementary Materials

Supplementary Materials S1: the pooled odds ratio of the association between fingernail trimming and cleanness and HIPPIs in Ethiopia. S2: the pooled odds ratio of the association between handwashing habits and HIPPIs in Ethiopia. S3: the pooled odds ratio of the association between age and HIPPIs in Ethiopia. S4: the pooled odds ratio of the association between open field defecation habit and HIPPIs in Ethiopia. S5: the pooled odds ratio of the association between eating raw, undercooked, contaminated, and leftover food and HIPPIs in Ethiopia. S6: the pooled odds ratio of the association between the level of education and HIPPIs in Ethiopia. S7: the pooled odds ratio of the association between the level of family income and HIPPIs in Ethiopia. S8: the pooled odds ratio of the association between the unprotected drinking water sources and HIPPIs in Ethiopia. S9: the pooled odds ratio of the association between playing with soil and HIPPIs in Ethiopia. S10: the pooled odds ratio of the association between the number of family size and HIPPIs in Ethiopia.
