# Supplementary material for: Prevalence and Associated Risk Factors of Human Intestinal Protozoan Parasitic Infections in Ethiopia: A Systematic Review and Meta-Analysis
Source: J Parasitol Res. 2020 Oct 5;2020:8884064. doi: 10.1155/2020/8884064 (PMC7556079; doi:10.1155/2020/8884064)
Supplement: Supplementary Materials — S1: the pooled odds ratio of the association between fingernail trimming and cleanness and HIPPIs in Ethiopia. S2: the pooled odds ratio of the association between handwashing habits and HIPPIs in Ethiopia. S3: the pooled odds ratio of the association between age and HIPPIs in Ethiopia. S4: the pooled odds ratio of the association between open field defecation habit and HIPPIs in Ethiopia. S5: the pooled odds ratio of the association between eating raw, undercooked, contaminated, and leftover food and HIPPIs in Ethiopia. S6: the pooled odds ratio of the association between the level of education and HIPPIs in Ethiopia. S7: the pooled odds ratio of the association between the level of family income and HIPPIs in Ethiopia. S8: the pooled odds ratio of the association between the unprotected drinking water sources and HIPPIs in Ethiopia. S9: the pooled odds ratio of the association between playing with soil and HIPPIs in Ethiopia. S10: the pooled odds ratio of the association between the number of family size and HIPPIs in Ethiopia. [file 8884064.f1.doc]

Fig. S 1 The pooled odd ratio of the association between finger nail trimming and cleanness and HIPPIs among different groups of people in Ethiopia.

Fig. S2 The pooled odds ratio of the association between hand washing habit and HIPPIs among different groups of people in Ethiopia.

**Fig. S3** The pooled odds ratio of the association between age and HIPPIs among study subjects in Ethiopia.

Fig. S4 The pooled odds ratio of the association between open field defecation habit and HIPPIs among different groups of people in Ethiopia.

Fig. S5 The pooled odds ratio of the association between eating raw, under cooked, contaminated and left over food and HIPPIs among different groups of people in Ethiopia.

Fig. S6 The pooled odds ratio of the association between level of education and HIPPIs among different groups of people in Ethiopia.

Fig. S7 The pooled odds ratio of the association level of family income and HIPPIs among different groups of people in Ethiopia.

Fig. S8 The pooled odds ratio of the association between unprotected drinking water source and HIPPIs among different groups of people in Ethiopia.

Fig. S9 The pooled odds ratio of the association between playing with soil and HIPPIs among different groups of people in Ethiopia.

**Fig. S10** The pooled odds ratio of the association between number of family size and HIPPIs among different groups of people in Ethiopia.
